# Graft glycocalyx degradation in human liver transplantation

**DOI:** 10.1371/journal.pone.0221010

**Published:** 2019-08-15

**Authors:** Arie Passov, Alexey Schramko, Heikki Mäkisalo, Arno Nordin, Sture Andersson, Eero Pesonen, Minna Ilmakunnas

**Affiliations:** 1 Department of Anesthesiology, Intensive Care and Pain Medicine, University of Helsinki and Helsinki University Hospital, Helsinki, Finland; 2 Transplantation and Liver Surgery Clinic, Abdominal Center, University of Helsinki and Helsinki University Hospital, Helsinki, Finland; 3 Children's Hospital, Pediatric Research Center, University of Helsinki and Helsinki University Hospital, Helsinki, Finland; Texas A&M University, UNITED STATES

## Abstract

**Objective:**

Ischaemia/reperfusion-injury degrades endothelial glycocalyx. Graft glycocalyx degradation was studied in human liver transplantation.

**Methods:**

To assess changes within the graft, blood was drawn from portal and hepatic veins in addition to systemic samples in 10 patients. Plasma syndecan-1, heparan sulfate and chondroitin sulfate, were measured with enzyme-linked immunosorbent assay.

**Results:**

During reperfusion, syndecan-1 levels were higher in graft caval effluent [3118 (934–6141) ng/ml, P = 0.005] than in portal venous blood [101 (75–121) ng/ml], indicating syndecan-1 release from the graft. Concomitantly, heparan sulfate levels were lower in graft caval effluent [96 (32–129) ng/ml, P = 0.037] than in portal venous blood [112 (98–128) ng/ml], indicating heparan sulfate uptake within the graft. Chondroitin sulfate levels were equal in portal and hepatic venous blood. After reperfusion arterial syndecan-1 levels increased 17-fold (P < 0.001) and heparan sulfate decreased to a third (P < 0.001) towards the end of surgery.

**Conclusion:**

Syndecan-1 washout from the liver indicates extensive glycocalyx degradation within the graft during reperfusion. Surprisingly, heparan sulfate was taken up by the graft during reperfusion. Corroborating previous experimental reports, this suggests that endogenous heparan sulfate might be utilized within the graft in the repair of damaged glycocalyx.

## Introduction

Glycocalyx is a layer of interacting proteoglycans, glycosaminoglycans and glycoproteins covering the luminal endothelial surface. It serves to sustains microvascular homeostasis and integrity. Intact glycocalyx regulates vascular tone and permeability, and inhibits blood coagulation. Glycocalyx also appears to control inflammatory responses as it regulates endothelial adhesion and transmigration of leukocytes and retains cytokines and chemokines within its heparan sulfate mesh [1,2].

Glycocalyx degradation occurs under pathophysiologic inflammatory conditions such as sepsis [3], trauma [4], and ischemia/reperfusion (IR) -injury [5–7]. IR-injury is inherent to liver transplantation. Graft preservation (cold ischemia) primes and activates Kupffer cells and sinusoidal endothelial cells to release reactive oxygen species (ROS) and inflammatory mediators and to express tissue factor upon reperfusion, resulting in direct cytotoxic effects, neutrophil recruitment into the liver, and local thrombin generation and intravascular coagulation [8]. Glycocalyx on the sinusoidal endothelium may be damaged not only by ROS, but also by matrix metalloproteinases (MMP), neutrophil-derived elastase and thrombin [1]. The altered glycocalyx structure and function may further exacerbate liver IR-injury by inducing sinusoidal vasoconstriction and tissue edema, by accelerating intravascular thrombosis, and by activating and recruiting innate immune cells to the graft [1,2,9].

Although elevated levels of heparan sulfate [2] and syndecan-1 [10], the biomarkers of glycocalyx degradation [11], have been reported in the systemic circulation both in clinical liver surgery with hepatoduodenal ligament clamping [2] and in liver transplantation in cirrhotic patients [10], neither of these studies addressed the potential IR-injury induced glycocalyx degradation within the liver.

We hypothesized that glycocalyx degradation occurs within the liver graft during reperfusion, and therefore studied plasma proteoglycan (syndecan-1) and glycosaminoglycan (heparan sulfate, chondroitin sulfate) kinetics across the liver graft in human liver transplantation. To this end, blood samples were obtained both from the portal vein (ingoing blood) and hepatic vein (outcoming blood) during and after liver graft reperfusion. As the aim of this study was to investigate the effects of IR-injury on the hepatic glycocalyx, we sought to eliminate the effects of end-stage liver disease on glycocalyx [10,12] by enrolling only patients undergoing preemptive liver transplantation for primary sclerosing cholangitis (PSC) [13,14] without cirrhosis and thus having normal liver function preoperatively.

## Materials and methods

### Patients

The study protocol was approved by the ethics committee in Helsinki University Hospital (Dnro 156/13/03/02/2014). After written informed consent, 10 PSC-patients undergoing orthotopic liver transplantation participated in the study (Table 1). All patients had a moderate suspicion of cholangiocarcinoma in their brush-cytology samples retrieved with endoscopic retrograde cholangiography (ERC). In addition, local progression of bile duct strictures supported the suspicion of cancer. Thus, the transplantation was performed to prevent dysplastic bile duct lesions from proceeding to invasive carcinoma. One patient had recurrent cholangitis with exacerbation prior to transplantation and thus higher bilirubin level that was reflected in higher Model for End-stage Liver Disease (MELD)- and Mayo risk scores. Of the 10 patients, 7 had also inflammatory bowel disease (6 ulcerating colitis, 1 Crohn’s disease). Immunosuppression was achieved with the combination of cyclosporin A, mycophenolate mofetil and methylprednisolone.

**Table 1:** Patient and graft characteristics.

Age (years)		49 (28–66)
Gender (male/female)		9/1
MELD-score		6 (6–13)
Child-Pugh score		5 (5–7)
Mayo risk score		-0.23 (-1.45–2.09)
Cold ischemic time (hours)		4.7 (3.5–9.7)
Time from the beginning of surgery until portal vein clamping (minutes)		174 (135–209)
Anhepatic time (minutes)		51 (44–80)
Time from portal vein declamping until hepatic artery declamping (minutes)		31 (21–47)
Perioperative bleeding (mL)		1600 (900–2600)
Graft steatosis		
	Grafts with any steatosis (n)	6
	Macrovesicular (%)	0 (0–20)
	Microvesicular (%)	0 (0–95)
Postoperative liver function		
	peak ALT (IU/L)	438 (213–2191)
	INR on day 7	1.2 (1.0–1.3)
	Bilirubin on day 7 (μmol/L)	17 (7–154)
	MEAF-score	2.84 (1.38–4.81)

Data are expressed as medians (range). MELD, Model for End-stage Liver Disease; ALT, alanine aminotransferase; INR, international normalized ratio; MEAF, Model for Early Allograft Function.

### Surgery and graft reperfusion

All grafts were retrieved from brain-dead donors and preserved with University of Wisconsin solution at 4°C. The surgical technique of the graft implantation and reperfusion was standardized. All transplantations were performed with cross-clamping of the inferior caval vein. Neither "piggy-back" technique nor veno-venous bypass was used. Before the anhepatic period, i.e. clamping of hepatic artery and portal vein and native supra- and infrahepatic caval vein, all patients were anticoagulated with 5000 IU heparin. Additional doses were given to maintain activated clotting time (ACT) at 200–300 seconds before the anhepatic period. During graft implantation, suprahepatic caval anastomosis was completed first. The infrahepatic caval and portal anastomoses were then partially completed. With the suprahepatic caval vein clamped, the graft was flushed via the portal vein first with 1000 mL of Ringer’s solution, followed by flushing with approximately 400 mL of portal venous blood. Both Ringer’s solution and portal venous blood were wasted from the infrahepatic caval anastomosis, blood constituting the graft caval effluent. Portal and infrahepatic caval vein anastomoses were completed and the caval and portal vein clamps were removed. Then the hepatic arterial anastomosis was completed and artery declamped. Finally, the biliary tract was reconstructed with a Roux-en-Y hepaticojejunostomy

Surgery was performed under balanced inhalational anesthesia. Vasoactive agents used were phenylephrine boluses and/or noradrenaline-infusion. Fluid therapy during liver transplantation was left to the discretion of the attending anesthesiologist. For data analysis, we recorded total amount and type of the fluids and blood products used during surgery.

### Blood samples

During liver transplantation, systemic arterial blood samples were collected after induction of anesthesia but before surgery (time point 1), immediately before reperfusion of the graft with portal blood (time point 2), 5 minutes after portal vein declamping (time point 3), and 5 minutes after hepatic artery declamping (time point 4). To assess changes across the graft, blood samples were obtained by puncture from both portal and hepatic veins at the following time points: during reperfusion (time point 2; at this time point the sample representing hepatic venous blood was drawn from the graft caval effluent), 5 minutes after portal vein declamping (time point 3), and 5 minutes after hepatic artery declamping (time point 4).

The volume of each sample was 10 mL. All samples were drawn into pyrogen-free syringes (BD Plastipak, Madrid, Spain) and then transferred into sodium citrate tubes (BD Vacutainer, Plymouth, UK) on melting ice at 0°C. Plasma was separated within 15 minutes by centrifugation at 2000g for 10 minutes and stored in aliquots at -80°C until the analyses were performed.

### Syndecan-1

Plasma syndecan-1 (human sCD138) levels were measured with a commercial enzyme-linked immunosorbent (ELISA) assay (Diaclone SAS, Besancon Cedex, France). Reproducibility coefficient of variation was 10.2% and assay sensitivity 4.94 ng/ml.

### Heparan sulfate

Plasma heparan sulfate levels were measured with a commercial ELISA assay (Elabscience Biotechnology Co.) for human heparan sulfate. Reproducibility coefficient of variation was <10% and assay sensitivity 0.188 ng/ml.

### Chondroitin sulfate

Plasma chondroitin sulfate levels were measured with a commercial ELISA assay (Abbexa Ltd, Cambridge, UK) for chondroitin sulfate. Reproducibility coefficient of variation was < 10% and assay sensitivity 0.188 ng/ml.

### Clinical assessment

Plasma alanine aminotransferase (ALT), international normalized ratio (INR), and bilirubin were measured daily as a part of the routine follow-up. For data analysis, we included the peak ALT value measured within 7 postoperative days, and INR and bilirubin levels measured at postoperative day 7 [15]. We also calculated the Model for Early Allograft Function (MEAF) -score from ALT, INR and bilirubin levels [16]. Graft steatosis was assessed from routine biopsies taken immediately before graft perfusion during the donor operation. The graft and patient outcome and complications within 30 days after transplantation was recorded.

### Statistical analysis

All statistical analyses were performed with SPSS version 22 software package (IBM Corporation, Armonk, New York, USA). Due to the small number of patients, nonparametric tests were used. To calculate the transhepatic gradient, portal vein value was subtracted from the caval effluent or hepatic vein value. Unpaired data were compared with the Mann-Whitney U-test, whereas paired data were compared with the Wilcoxon signed rank test. Time-dependent changes were evaluated by Friedman’s test with post hoc Wilcoxon signed rank test with Holm’s correction for multiple comparisons. Bivariate correlations were evaluated by the Spearman rank correlation. The α-level was 0.05 for all statistical tests. Data are expressed as medians with ranges.

## Results

### Clinical outcome

Perioperative bleeding was relatively minor (Table 1). In addition to Ringer’s acetate used for perioperative fluid therapy in all patients [5750 (range 4500–6700) mL], seven patients received 4% albumin [800 (range 400–1400) mL] and, four patients received fresh frozen plasma (FFP) [400 (range 400–800) mL] according to the clinical judgment of the anesthesiologist. Only one patient received packed red cells (3 units).

Five patients had significant postoperative complications. Four patients underwent relaparotomy, one due to biliary leakage, three due to postoperative bleeding. One of the patients with postoperative bleeding requiring laparotomy on postoperative day 5 had undergone dialysis for acute kidney injury before the bleeding. One patient had an unspecified infection, treated with antibiotics from postoperative day 8 onwards. Despite these complications, postoperative liver function was good in all recipients (Table 1). Both patient and graft 30-day survival were 100%. Three patients developed acute rejection, treated with steroids, during the 30-day follow-up. Despite the preoperative suspicion of cancer none of the patients had cholangiocarcinoma in their explanted livers. None of the explanted livers demonstrated significant fibrosis.

### Syndecan-1

**Fig 1:**
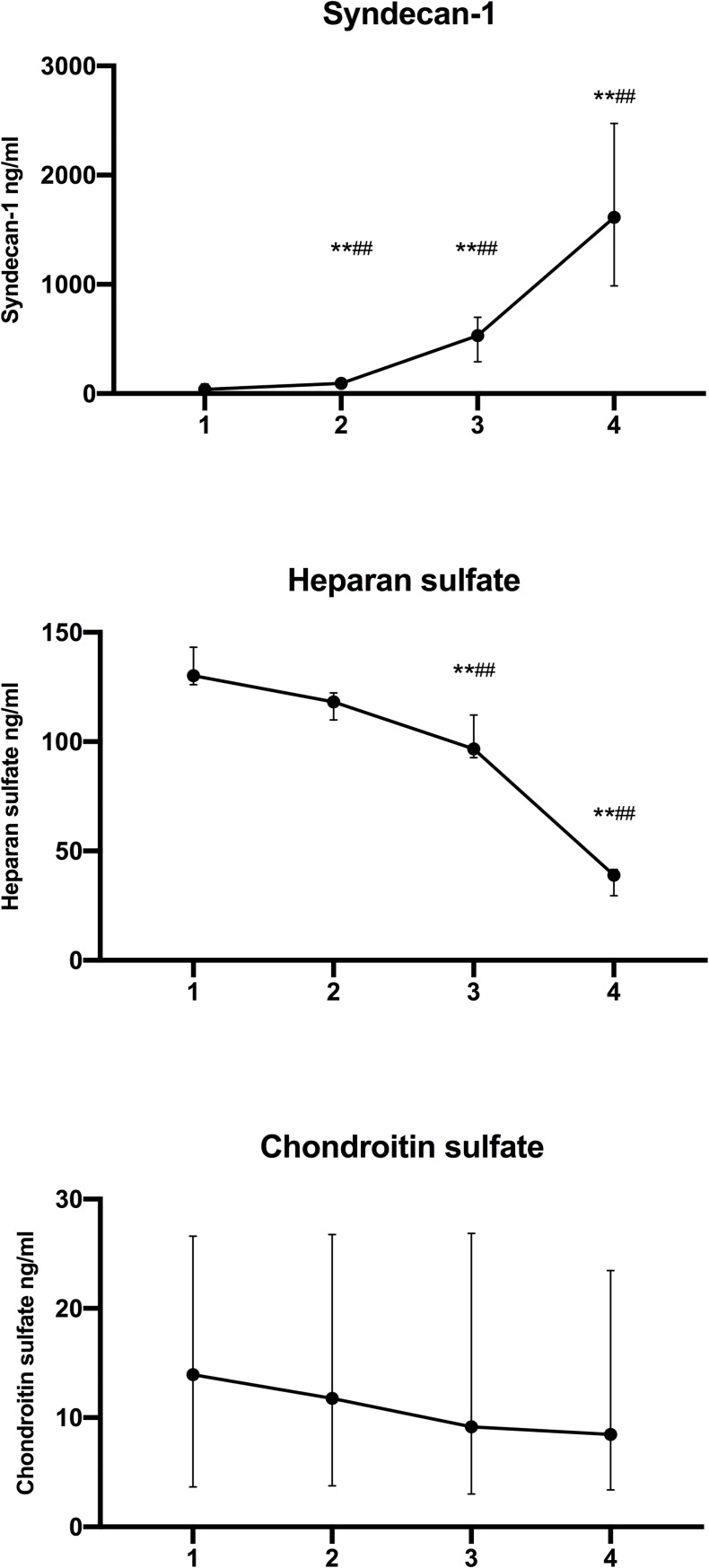
Systemic concentrations of syndecan-1, heparan sulfate and chondroitin sulfate preoperatively (1), before reperfusion (2), 5 min after portal vein declamping (3), and 5 min after hepatic artery declamping (4). Data are depicted as median and interquartile range. ** P < 0.01 for vs preoperatively (1) and ^##^ P < 0.01 for vs before reperfusion (2) (Wilcoxon Signed Rank test).

**Fig 2:**
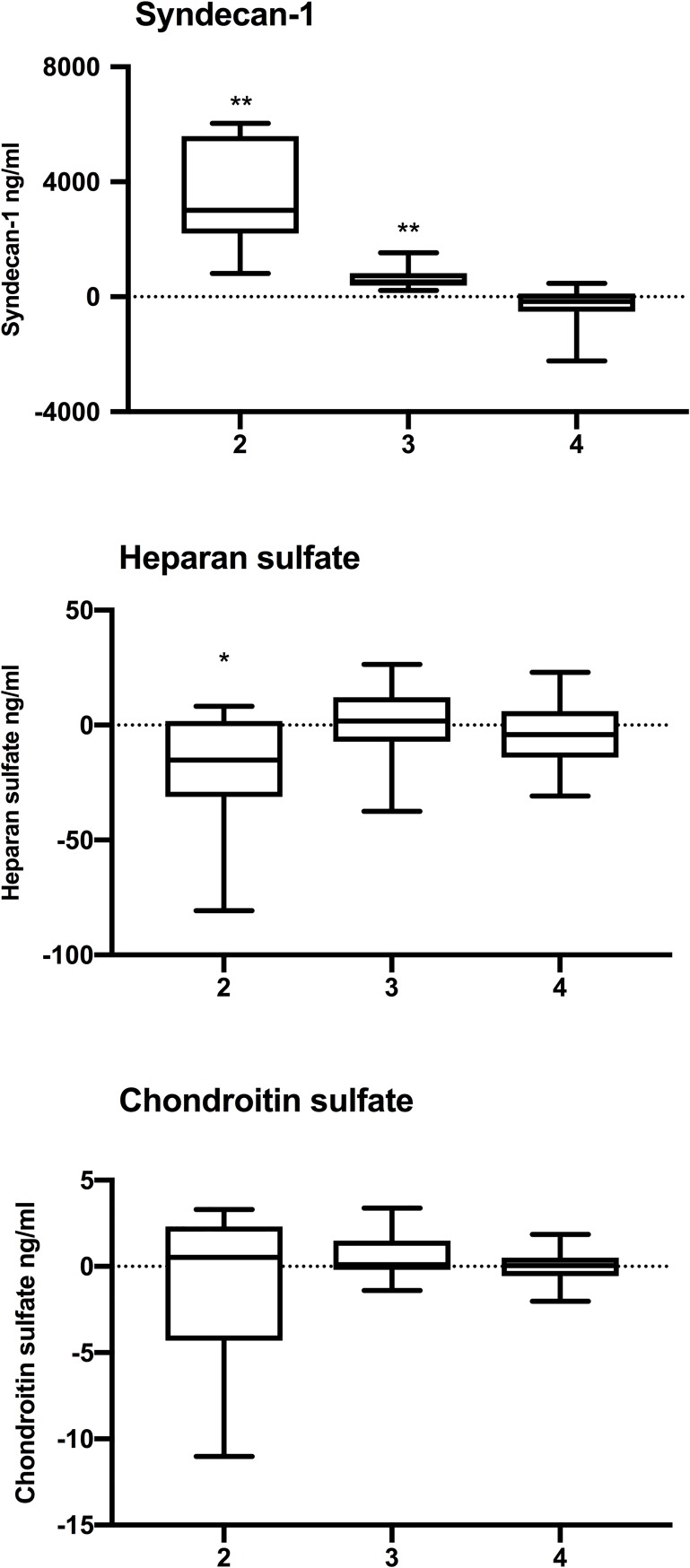
Transhepatic gradients [(caval effluent or hepatic vein)–(portal vein)] of syndecan-1, heparan sulfate and chondroitin sulfate at reperfusion (2), 5 minutes after portal vein declamping (3) and 5 minutes after hepatic artery declamping (4). * P < 0.05 and ** P < 0.01 for hepatic vs portal vein (Wilcoxon Signed Rank test).

Plasma syndecan-1 levels in the systemic circulation increased during surgery, significantly so after graft reperfusion (*P* 0.001 Friedman’s test; Fig 1). During reperfusion, plasma syndecan-1 levels were significantly higher in the caval effluent as compared to portal venous blood, indicating syndecan-1 outflow from the liver graft (Table 2, Fig 2). This syndecan-1 outflow continued at 5 min after portal vein declamping (Table 2, Fig 2).

**Table 2:** Plasma syndecan-1, heparan sulfate and chondroitin sulfate concentrations during graft reperfusion.

		Syndecan-1 (ng/ml)	Heparan sulfate (ng/ml)	Chondroitin sulfate ng/ml)
Reperfusion				
	Portal vein	101 (75–121)	112 (98–128)	12.7 (0.95–76.1)
	Caval effluent	3118 (934–6141)	96 (32–129)	10.4 (0.5–73.1)
	Transhepatic gradient^1^	3013 (814–6036)**	-15 (-81–8)*	0.5 (-11.0–3.3)
5 min after portal vein declamping				
	Portal vein	379 (202–830)	102 (73–126)	6.9 (0.5–40.6)
	Hepatic vein	1038 (202–2207)	102 (74–128)	7.7 (0.6–44.0)
	Transhepatic gradient	514 (220–1528)**	2 (-37–26)	0.1 (-1.4–3.4)
5 min after hepatic artery declamping				
	Portal vein	1565 (515–3590)	36 (31–55)	8.3 (0.25–37.7)
	Hepatic vein	1294 (583–3590)	30 (21–56)	7.6 (0.3–39.6)
	Transhepatic gradient	-165 (-2236–468)	-4 (-31–23)	0.1 (-2.0–1.9)

Data are expressed as median (range).

^1^ [(caval effluent or hepatic vein)–(portal vein)].

* P <0.05

** P < 0.01 (hepatic vs portal vein, Wilcoxon Signed Rank test).

### Heparan sulfate

Plasma heparan sulfate levels in the systemic circulation decreased during surgery (*P* < 0.001, Friedman’s test; Fig 1). During reperfusion, plasma heparan sulfate levels were significantly lower in the caval effluent as compared to portal venous blood, indicating heparan sulfate uptake within the liver graft (Table 2, Fig 2).

### Chondroitin sulfate

Plasma chondroitin sulfate levels in systemic circulation decreased slightly during surgery, but the change was statistically not significant (Fig 1). Chondroitin sulfate levels were similar in portal blood and hepatic venous blood (Table 2, Fig 2).

### Glycocalyx degradation and clinical parameters

Neither syndecan-1, heparan sulfate nor chondroitin sulfate levels correlated with graft steatosis, cold ischemic time, postoperative liver function or MEAF-score. Likewise, markers of glycocalyx degradation did not correlate with the amount or type of fluids administered perioperatively.

## Discussion

The main finding of this study, somewhat surprisingly, is heparan sulfate sequestration within the liver graft during reperfusion. Also, concomitant decrease in systemic heparan sulfate levels occured after reperfusion. Still, this heparan sulfate uptake was accompanied by extensive release of proteoglycan syndecan-1 from the liver graft during reperfusion, together with a rapid increase in systemic syndecan-1 levels after reperfusion.

In accordance with our hypothesis, changes in syndecan-1 concentrations indicate that glycocalyx degradation within the liver graft occurred during graft reperfusion. The results regarding the release of syndecan-1 from the liver graft are in line with those of Snoeijs et al [7], who demonstrated an outflow of syndecan-1 from the graft in human kidney transplantation. The marked syndecan-1 efflux from the liver graft may reflect glycocalyx degradation occurring both during graft cold preservation and reperfusion. In experimental models of liver IR-injury, sinusoidal endothelial cells appear particularly sensitive to cold ischemia [17] and glycocalyx damage in the sinusoids has been described during cold preservation [18]. Thus, the syndecan-1 efflux during reperfusion demonstrated in our patients potentially reflects a washout of glycocalyx degradation products accumulated in the graft during cold preservation. In addition to simple washout, the syndecan-1 efflux from the graft likely indicates exacerbated damage to the sinusoidal endothelium during graft reperfusion. Indeed, ROS released from sinusoidal endothelial cells, Kupffer cells and neutrophils during reperfusion [2,19] can directly induce glycosaminoglycan fragmentation. ROS also trigger endothelial cells, leukocytes and platelets to release heparanase, an enzyme degrading both heparan sulfate and syndecan-1 [20]. Furthermore, a rapid and extensive hepatic release of MMPs and elastase occurs during graft reperfusion in human liver transplantation [21] and these proteolytic enzymes degrade components of glycocalyx [1,22]. In our patients, syndecan-1 release from the graft was reflected in syndecan-1 levels in the systemic circulation, which increased significantly after graft reperfusion, and increased continuously towards the end of the operation.

Concurrently with the increasing syndecan-1 levels in the systemic circulation, heparan sulfate levels decreased substantially during surgery. Our finding is surprising and contradicts the results from previous clinical studies. In patients undergoing vascular [6] or cardiac surgery [6,23], both biomarkers of glycocalyx degradation exhibit similar kinetics, i.e. heparan sulfate levels increase in parallel with those of syndecan-1. Furthermore, during liver surgery with hepatoduodenal ligament clamping, systemic heparan sulfate levels increase, although this increase seemed to relate to surgery *per se* and not IR-injury [2]. In our patients, the decrease in systemic heparan sulfate levels was marked particularly after the reperfusion of the liver graft. Within an hour after graft reperfusion (at 5 min after hepatic artery declamping), the median heparan sulfate level in the systemic circulation had decreased to a third as compared to pre-reperfusion levels. Although momentary heparan sulfate sequestration within the liver, measured as transhepatic difference in heparan sulfate levels, could be demonstrated only during reperfusion, it seems likely that the continuously decreasing systemic heparan sulfate levels reflected ongoing hepatic heparan sulfate uptake. Due to the surprising results in heparan sulfate kinetics, we measured also chondroitin sulfate levels, since it is the second most abundant glycosaminoglycan in the glycocalyx after heparan sulfate [24]. However, neither hepatic washout nor uptake of chondroitin sulfate was observed.

We can only speculate the pathophysiologic processes of hepatic heparan sulfate uptake. We propose that heparan sulfate might be utilized in the reperfused graft in an attempt to repair the damaged glycocalyx. Indeed, in human kidney transplantation, after the initial degradation, glycocalyx thickness increased significantly within 30 min after reperfusion [7]. Likewise, in rodent hemorrhagic shock, volume resuscitation with fresh frozen plasma, but not with lactated Ringer’s solution, restored glycocalyx within two hours [25,26]. These in vivo results indicate that damaged glycocalyx may be repaired very rapidly (within the sampling time range of the present study) through endogenous processes. Furthermore, in experimental models with chemical glycocalyx degradation, intravascular administration of exogenous heparan sulfate restores glycocalyx [27,28]. Because we did not take graft biopsies nor intravital microscopy for direct evaluation of glycocalyx thickness graft heparan sulfate uptake as a reflection of acute glycocalyx reparation remains speculative to this date.

In our highly selected patient cohort, either syndecan-1 release, that reflects damaged glycocalyx, or potential reparative heparan sulfate uptake, did not translate into clinical outcomes. However, preventing glycocalyx degradation during graft preservation and reperfusion could offer a strategy to improve clinical outcomes in liver transplantation, especially with regard to marginal grafts. The seemingly most feasible method for protecting graft glycocalyx during preservation is to supplement the preservation solution with substances known to inhibit glycocalyx degradation under inflammatory conditions, for example steroids [29–31], serine protease inhibitors antithrombin [32] and tranexamic acid [33,34], heparin and heparinoids [28,35,36] or sphingosine-1-phospate [28,37]. None of the current commercially available preservation solutions contain these substances [38]. Where machine perfusion is available, replacing gelatin [39] with albumin or FFP [25,26,40], or supplementing the perfusion solution with any one of the above-mentioned substances might be feasible. Alternatively, glycocalyx degradation might be alleviated by graft flushing with FFP or albumin [25,26,40] immediately prior to reperfusion.

Our study has several strenghts. First, the surgeons took the blood samples by direct needle puncture from the portal and hepatic veins. Thus, the obtained results reflect real concentration differences across the reperfused liver. Second, our study cohort was homogenous. As patients with chronic liver disease have elevated syndecan-1 [10] and heparan sulfate [12] levels, we chose to eliminate this confounding factor by enrolling a homogenous group of patients undergoing liver transplantation for PSC with premalignant biliary lesions [13,14] and thus having normal preoperative liver function. The chosen patient selection was reflected in the low preoperative (MELD) score of median 6.

There are limitations to this study. First, given the small number of highly selected patients and non-steatotic grafts with short cold ischemic times, and thus excellent postoperative liver function with a median MEAF-score of 2.84 [16], the lacking correlations between syndecan-1, heparan sulfate or chondroitin sulfate levels during reperfusion and the subsequent liver function are not surprising. Thus, our findings cannot be generalized into cirrhotic patients or marginal grafts. Second, seven of the ten patients had inflammatory bowel disease, which may have slightly increased the baseline syndecan-1 levels [41]. Third, the levels of glycocalyx degradation makers might have been affected by the unstandardized fluid therapy during transplantation. Indeed, the administration of albumin in seven patients and FFP in four patients might have affected syndecan-1 levels (25,26,39). Fourth, during the anhepatic period, all patients were anticoagulated with heparin and received a standard dose of methylprednisolone prior to graft reperfusion, both of which may alleviate glycocalyx degradation [29,30,35] and thus might potentially have affected syndecan-1, heparan sulfate and chondroitin sulfate levels in our patients.

Taken together, we demonstrated graft glycocalyx degradation during reperfusion in human liver transplantation in a selected patient cohort with normal liver and hemostatic function. The extensive syndecan-1 release from the graft was associated with concomitant heparan sulfate uptake into the graft, suggesting heparan sulfate can be utilized within the graft in pathophysiologic processes. To the best of our knowledge, this is the first in vivo observation of endogenous heparan sulfate uptake in a clinical context. In the light of previous experimental [25–28] and clinical [7] literature, we speculate that graft heparan sulfate uptake in our study might reflect endogenous reparative processes to mend the damaged glycocalyx. Our findings call for both experimental and clinical studies to delineate the actual mechanisms of glycocalyx degradation and reparation in cold ischemic IR-injury. Likewise, future studies in larger patient cohorts are needed to explore the clinical significance of graft glycocalyx degradation in human liver transplantation.
